# Support for a Photoprotective Function of Winter Leaf Reddening in Nitrogen-Deficient Individuals of *Lonicera japonica*

**DOI:** 10.3390/molecules191117810

**Published:** 2014-11-03

**Authors:** Kaylyn L. Carpenter, Timothy S. Keidel, Melissa C. Pihl, Nicole M. Hughes

**Affiliations:** Department of Biology, High Point University, University Station 3591, High Point, NC 27262, USA

**Keywords:** anthocyanin, evergreen, *Lonicera japonica*, nitrogen, photosynthesis, winter

## Abstract

Plants growing in high-light environments during winter often exhibit leaf reddening due to synthesis of anthocyanin pigments, which are thought to alleviate photooxidative stress associated with low-temperature photoinhibition through light attenuation and/or antioxidant activity. Seasonal high-light stress can be further exacerbated by a limited photosynthetic capacity, such as nitrogen-deficiency. In the present study, we test the following hypotheses using three populations of the semi-evergreen vine *Lonicera japonica*: (1) nitrogen deficiency corresponds with reduced photosynthetic capacity; (2) individuals with reduced photosynthetic capacity synthesize anthocyanin pigments in leaves during winter; and (3) anthocyanin pigments help alleviate high-light stress by attenuating green light. All populations featured co-occurring winter-green and winter-red leafed individuals on fully-exposed (high-light), south-facing slopes in the Piedmont of North Carolina, USA. Consistent with our hypotheses, red leaves consistently exhibited significantly lower foliar nitrogen than green leaves, as well as lower total chlorophyll, quantum yield efficiency, carboxylation efficiency, and photosynthesis at saturating irradiance (A_sat_). Light-response curves measured using ambient sunlight *versus* red-blue LED (*i.e*., lacking green wavelengths) demonstrated significantly reduced quantum yield efficiency and a higher light compensation point under sunlight relative to red-blue LED in red leaves, but not in green leaves, consistent with a (green) light-attenuating function of anthocyanin pigments. These results are consistent with the hypothesis that intraspecific anthocyanin synthesis corresponds with nitrogen deficiency and reduced photosynthetic capacity within populations, and support a light-attenuating function of anthocyanin pigments*.*

## 1. Introduction

Anthocyanins are vacuolar, flavonoid pigments synthesized via the shikimic acid pathway that impart red to purplish colors in plant tissues [1]. Of special interest to plant physiologists is the synthesis of anthocyanin pigments in photosynthetic tissues during periods of high-light stress, which may be defined generally as seasons, ontogenetic stages, and/or environmental conditions corresponding with an imbalance of light capture relative to energy processing (for reviews see [1,2,3]). For example, anthocyanin synthesis has been observed under high light in combination with: cold temperatures [1,4,5], drought stress [6,7,8], leaf development [9,10,11,12], and senescence [13,14]. However, the functional significance of leaf reddening remains a matter of debate (discussed in further detail below; for reviews see [5,14,15]). Furthermore, why some individuals or species synthesize red pigments, while others do not, is also not yet fully understood [5,16,17,18].

There are currently two functional explanations for anthocyanin synthesis in leaves—photoprotection and ecological defense. According to the photoprotection hypothesis, anthocyanins protect photosynthetic tissues vulnerable to high-light stress through antioxidant activity, and/or by intercepting green quanta, thereby alleviating excess chlorophyll excitation pressure in underlying cells [1,2,3]. According to the ecological defense hypothesis, anthocyanins function to reduce damage by potential herbivores or pathogens by either: (a) reducing visibility to herbivores lacking a red photoreceptor (*i.e.*, camouflage); (b) signaling low leaf quality (e.g., high investment in chemical defenses, low nitrogen content) [10,19,20,21]; (c) undermining herbivorous insect camouflage [22]; and/or (d) inhibiting fungal growth [23,24]. Because plant-insect interactions are generally less frequent during the winter, we focus here on the putative photoprotective function of anthocyanin pigments, as this function seems most directly relevant to the high-light, cold temperature conditions in which winter-leaf reddening frequently occurs [5].

During winter, high-light in combination with cold temperatures results in excess energy capture by chlorophylls relative to (reduced) energetic demands of the Calvin cycle [25]. The resulting photooxidative damage and associated photoinhibition of photosynthesis further reduce carbon gain, and plants have evolved photoprotective strategies to alleviate this imbalance accordingly. Such strategies include: increases in xanthophyll-cycle pigments, increased conversion of violaxanthin to zeaxanthin, selective degradation and/or sustained-phosphorylation of D1/D2 protein and whole PSII cores, increased antioxidant pools, vertical leaf orientation, and/or synthesis of photoprotective anthocyanin pigments [5,26,27,28,29,30,31,32,33]. As would be expected, relative engagement of photoprotection has been shown to be inversely correlated with energy processing capacity [34,35]. Hence, anthocyanin synthesis might be expected to occur in individuals or species with diminished capacity for photosynthesis and⁄or energy dissipation.

Recent studies on intraspecific populations featuring co-occurring red and green individuals have demonstrated that red-leafed individuals tend to exhibit symptoms of photosynthetic inferiority relative to co-occurring green-leafed individuals, including lower leaf nitrogen, lower photosynthetic capacity, and greater photoinhibition of photosynthesis [36,37,38,39,40,41,42]. Because foliar nitrogen levels are directly correlated with molecular and enzymatic pools involved in photosynthesis, including Rubisco, chlorophyll, and chlorophyll binding protein [43,44,45], nitrogen deficiency not only reduces a plant’s capacity for light capture and processing [46], but also increases its need for photoprotection [34,35]. The photosynthetic-inferiority hypothesis posits that individuals suffering from physiological limitations to energy processing, such as nitrogen deficiency, should synthesize anthocyanins as a means of alleviating this photosynthetic imbalance [5,39,42,47]. To date, this idea has only been tested in a few species, and evidence linking nitrogen deficiency, photosynthetic capacity, anthocyanin production, and photoprotection all within an individual study system are sparse in the literature.

The objective of this study was to test the photosynthetic-inferiority hypothesis for leaf reddening using co-occurring red and green populations of Japanese honeysuckle, *Lonicera japonica* Thunb. *Lonicera japonica* is a non-native, semi-evergreen vine that is invasive to the USA, that often synthesizes anthocyanins in sun-exposed leaves during winter under high-light conditions [16]. We utilize three separate, high-light field sites in the Piedmont of North Carolina featuring co-occurring winter-red (anthocyanic) and winter-green (acyanic) populations of *L. japonica* to test the hypothesis that red-leafed individuals correspond with lower leaf nitrogen content and associated photosynthetic deficiencies (e.g., lower carboxylation efficiency, reduced chlorophyll content, reduced capacity for photosynthesis) relative to co-occurring green individuals. We further test whether light attenuation by anthocyanin results in physiologically significant reductions in green light absorption in red-leafed individuals, which would support a photoprotective function for leaf reddening.

## 2. Results and Discussion

### 2.1. Leaf Nitrogen

**Figure 1:**
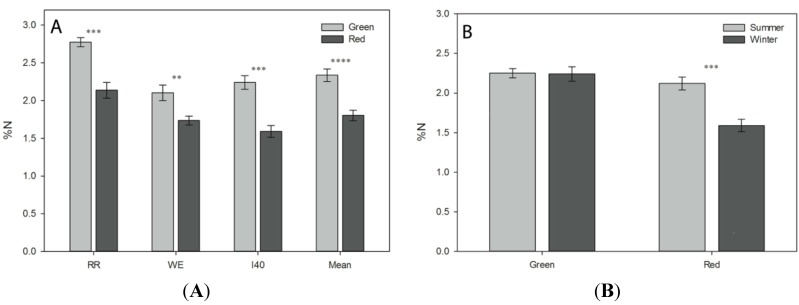
Mean nitrogen content in red *versus* green *L. japonica* leaves. (**A**) Winter mean percent nitrogen content (±SE) at RR (n_green_ = 5, n_red_ = 5), WE (n_green_ = 7, n_red_ = 6), I40 (n_green_ = 6, n_red_ = 6), and combined means from all sites. Significant differences between red and green leaves denoted by asterisks (* *p* < 0.05; ** *p* < 0.01, *** *p* < 0.001, and **** *p* < 0.0001); (**B**) Mean percent leaf nitrogen (±SE) from leaf tissues collected at I40 during summer (n_winter-green_ = 6, n_winter-red_ = 6) and winter (n_green_ = 6, n_red_ = 6).

Winter-red individuals at RR, WE and I40 had significantly (23% on average) lower leaf N content than winter green-leafed individuals (*p* < 0.01 at WE, *p* < 0.001 at RR and I40 and *p* < 0.0001 when combined; Figure 1A). When individual sites were compared, winter leaf N content was highest at the RR site, with mean N content of 2.8% and 2.1% for green and red leaves respectively. At WE, green leaves had a mean N content of 2.1% during winter, and red leaves, 1.74%; I40 green leaves had a mean N content of 2.24%, and red leaves, 1.59% during winter. When summer (all green) leaves were compared from I40, leaves on the winter-green side of the embankment continued to exhibit higher average N content relative to leaves on the winter-red side, though this difference was only marginally significant (*p* = 0.12). Leaves from the winter-green side of the embankment showed no significant difference in foliar N between winter and the following summer (*p* = 0.96), while the leaves from the winter-red side red exhibited significant increases in percent nitrogen between winter and the following summer (*p* < 0.001; Figure 1B).

### 2.2. Chlorophyll Content

**Figure 2:**
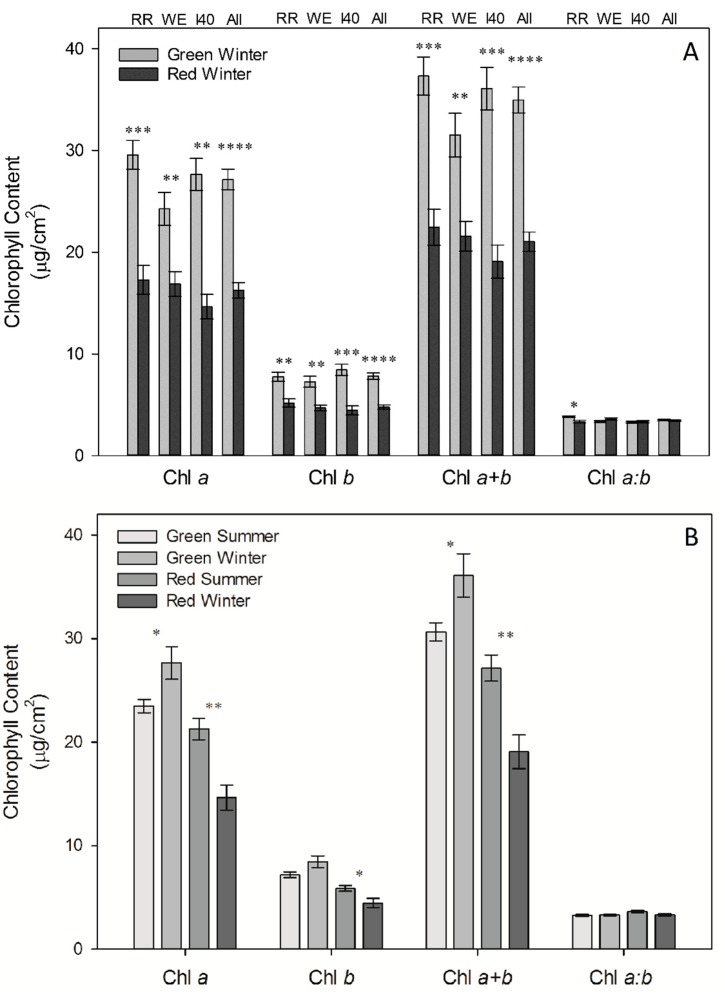
(**A**) Mean chlorophyll content per unit leaf area during winter for green (*n* = 5) compared to red (*n* = 5) leaves ± SE at each site for RR, WE, I40, and combined sites (see labels at top of figure); (**B**) Mean summer chlorophyll content for winter-red (*n* = 6) and winter-green (*n* = 6) portions of I40 site compared with winter chlorophyll content (*n =* 5 for both sides) ± SE. Significant differences between red *versus* green leaves (A); and summer *versus* winter leaves (B) denoted by asterisks (* *p* < 0.05; ** *p* < 0.01, *** *p* < 0.001, and **** *p* < 0.0001).

Red leaves exhibited consistently lower (40% on average) total chl during winter compared to co-occurring green leaves (*p* < 0.01 for WE, *p* < 0.001 at RR and I40, combined sites *p* < 0.0001 see Figure 2A). Trends in chl *a/b* ratios were less consistent between sites (Figure 2A). Chl *a/b* was significantly lower in red leaves relative to green during winter at RR (*p* < 0.05), but there were no statistically significant differences at either WE or I40. Analysis of combined winter data from all sites showed no significant difference in chl *a/b* between winter-red and winter-green leaves.

Leaves collected during summer from the winter-green side of the I40 embankment contained significantly higher total chl per unit leaf area and lower chl *a/b* than leaves on the winter-red side (*p* < 0.05 for both), consistent with trends observed at this site the previous winter (Figure 2B). However, differences in total chl were much smaller in magnitude than the values obtained during winter. When comparing summer *versus* winter total chl at I40 (Figure 2B), leaves on the winter-green side of the embankment exhibited significantly (18%) higher total chl content during winter relative to the summer (*p* < 0.05). However, the opposite was observed in leaves on the red-leafed side of the embankment, where leaves exhibited significant increases (42%) in chlorophyll content during summer relative to the previous winter (*p* < 0.01, Figure 2B). Chlorophyll *a/b* ratios were slightly lower in the winter-red leaves during winter than summer (*p* < 0.1), but winter-green leaves showed no notable differences.

### 2.3. Photosynthetic Gas Exchange

#### 2.3.1. Diurnal Measurements

**Figure 3:**
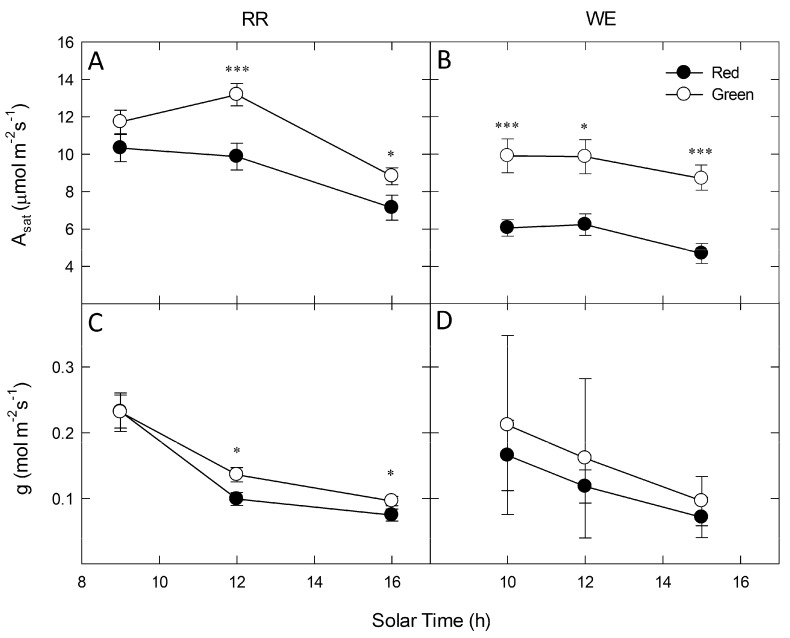
Diurnal measurements of photosynthetic gas exchange for co-occurring red leaves (closed circles) and green leaves (open circles) under saturating red/blue LED irradiance. Panels (**A**) and (**B**) show photosynthesis at saturating irradiance (A_sat_) and (**C**) and (**D**) show stomatal conductance (g) at RR (left column) and WE (right column). Points represent means of 3–15 individuals of each color ± SE. Significant differences between red and green leaves at each time point denoted by asterisks (* *p* < 0.05; ** *p* < 0.01, *** *p* < 0.001).

Diurnal photosynthetic gas exchange measurements at both fields sites (Figure 3) showed significantly reduced photosynthesis in red leaves relative to green under saturating red/blue LED irradiance throughout the day, with the only exception being the early morning measurement at the RR site (Figure 3A, RR: *p* < 0.001 at 1200, *p* < 0.05 at 1600, Figure 3B WE: *p* < 0.001 at 1000 and 1500, *p* < 0.05 at 1200). In general, photosynthesis tended to decrease in all plants during the day, corresponding with declines in leaf stomatal conductance to water vapor (g). On average, green leaves tended to have higher g than red-leaves at both sites, however, this difference was only significant in two measurements made at the RR site (*p* < 0.05 for midday and 1600, Figure 3C).

#### 2.3.2. Light-Response Curves

**Figure 4:**
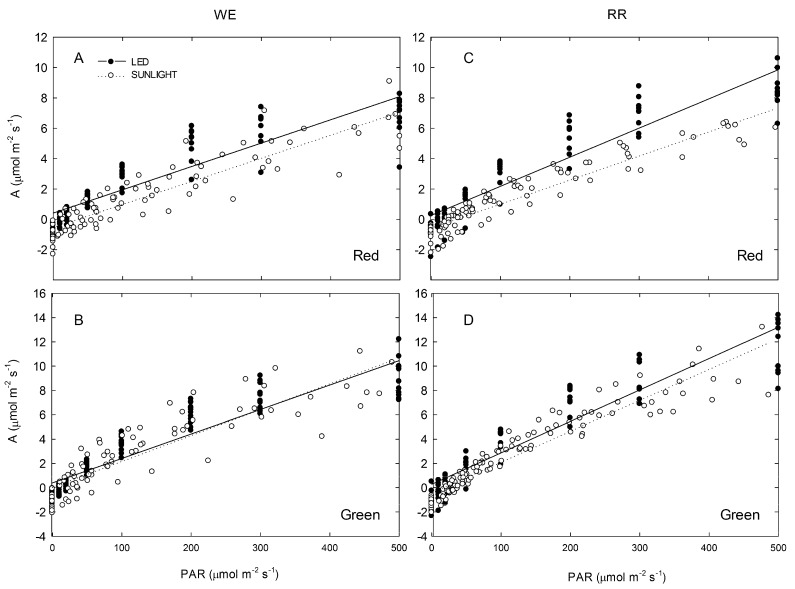
Linear (light-dependent) portion of light response curves measured under red/blue LED (closed symbols, solid line) *versus* ambient sunlight (open symbols, dashed line) at WE (left column) and RR (right column). (**A**) Red leaves at WE, n_LED_ = 10 n_Sun_ = 10; (**B**) Green leaves at WE, n_LED_ = 12 n_Sun_ = 9; (**C**) Red leaves at RR, n_LED_ = 9 n_Sun_ = 10; (**D**) Green leaves at RR, n_LED_ = 9 n_Sun_ = 13. All measurements made during winters of 2011–2013.

Light-response curves derived using red/blue LED *versus* ambient sunlight allowed for comparison of photosynthetic parameters with and without interference by the (green-light absorbing) anthocyanic layer (Figure 4, Table 1). Red leaves showed significantly reduced QYE (30% lower on average) under ambient sunlight relative to red/blue LED measurements at both field sites (WE *p* < 0.05, RR *p* < 0.001, combined *p* < 0.0001; Figure 4A,C). Additionally, significantly (180%) more PAR was required to reach LCP under ambient sunlight than under LED (WE and RR *p* < 0.05, combined *p* < 0.001). For green leaves, significant (albeit less dramatic) differences in QYE and LCP were observed under LED *versus* sunlight at RR (*p* < 0.05 for both; Figure 4D), but no significant differences were observed at WE (Figure 4B). Upon analyzing combined site data for winter-green leaves, no difference was found in the QYE between sunlight and LED light sources, though significantly more (68%) light was required to reach LCP under ambient sunlight compared to LED (*p* < 0.05). Both red and green leaves at RR had significantly greater photosynthesis at saturating irradiance (A_sat_) under LED relative to ambient sunlight (*p* < 0.01 for both), while no differences were found at WE for either red or green leaves (Table 1). When data from both field sites were combined, A_sat_ did not significantly differ when LED or sunlight was used as a saturating light source in red leaves, though green leaves had significantly (14%) higher A_sat_ under LED light (*p* < 0.05). Dark respiration measurements made following red/blue LED *versus* sunlight light response curves did not significantly differ in green leaves at either site, or in red leaves at RR; however, in red leaves at WE, DR was significantly lower (*i.e.*, greater respiration) following measurements made with ambient sunlight relative to measurements made with the red/blue LED (*p* < 0.05).

**Table 1:** Data derived from light response curves for red *versus* green leaves measured during winters of 2011–2013. Data are means derived during winter at West End (WE) and Railroad (RR) field sites, using either red/blue LED as a light source, or ambient sunlight. Data include: dark respiration rate (DR), light compensation point (LCP), quantum yield efficiency (QYE), and photosynthesis under saturating irradiance (A_sat_). Asterisks denote statistical significance between means (±SE) of red and green leaves at each site (* *p* < 0.05, ** *p* < 0.01, *** *p* < 0.001, **** *p* < 0.0001).

Red/Blue LED					
	*n*	DR (μmol·m^−2^·s^−1^)	LCP (μmol·m^−2^·s^−1^)	QYE	A_sat_ (μmol·m^−2^·s^−1^)
Red WE	10	−0.603 ± 0.27	14.7 ± 8.9	0.0350 ± 0.0056 **	7.88 ± 1.7 **
Green WE	12	−0.870 ± 0.39	15.6 ± 8.0	0.0442 ± 0.0076	11.1 ± 2.4
Red RR	9	−0.697 ± 0.76	18.0 ± 20	0.0362 ± 0.0080 *	11.4 ± 2.1 **
Green RR	9	−1.03 ± 0.83	18.9 ± 15	0.0504 ± 0.013	16.4 ± 3.7
Red AVG	19	−0.648 ± 0.55	16.3 ± 15	0.0356 ± 0.0067 ***	9.53 ± 2.6 ****
Green AVG	21	−0.939 ± 0.61	17.0 ± 11	0.0469 ± 0.010	13.4 ± 4.0
**Ambient Sunlight**					
Red WE	10	−1.12 ± 0.71	49.6 ± 39	0.0255 ± 0.0094 **	8.74 ± 2.4 *
Green WE	9	−1.20 ± 0.82	26.7 ± 23	0.0502 ± 0.023	11.0 ± 1.7
Red RR	10	−1.04 ± 0.52	41.9 ± 25	0.0241 ± 0.0045 ****	8.96 ± 1.4 ***
Green RR	13	−1.28 ± 0.36	29.8 ± 9.0	0.0398 ± 0.0049	12.4 ± 2.7
Red AVG	20	−1.08 ± 0.60	45.8 ± 32 *	0.0248 ± 0.0072 ****	8.85 ± 1.9 ****
Green AVG	22	−1.24 ± 0.57	28.6 ± 16	0.0440 ± 0.015	11.8 ± 2.4

Statistical analyses were also used to compare photosynthetic parameters for red *versus* green leaves within each field site, under the same type of light (rather than between types of light). No significant differences were observed in dark respiration (DR) between red and green leaves at either field site, or when site values were combined (*p* > 0.4 for all; Table 1). PAR intensities required to reach LCP also did not differ for red *versus* green leaves at either site under red/blue LED or ambient sunlight (*p* > 0.4 for both; Table 1). However, when site data were combined, LCP was 60% higher in red leaves, but only under ambient sunlight (*p* < 0.05). Green leaves exhibited significantly higher QYE and A_sat_ relative to red leaves at both sites under red/blue LED (QYE: WE *p* < 0.01, RR *p* < 0.05; A_sat_: *p* < 0.01 at both sites; Table 1). When data from both field sites were combined, QYE values were an average of 32% higher in green *versus* red leaves, and A_sat_ was 40% higher in green *versus* red under red/blue LED (*p* < 0.001 for both, Table 1). Under sunlight, mean QYE and A_sat_ were also significantly higher in green leaves compared to red at both sites (77% and 33% higher on average, respectively; QYE: WE *p* < 0.01, RR and combined sites *p* < 0.0001; A_sat_: WE *p* < 0.05, RR *p* < 0.001, combined sites *p* < 0.0001, Table 1).

#### 2.3.3. A/C_i_ Curves

**Figure 5:**
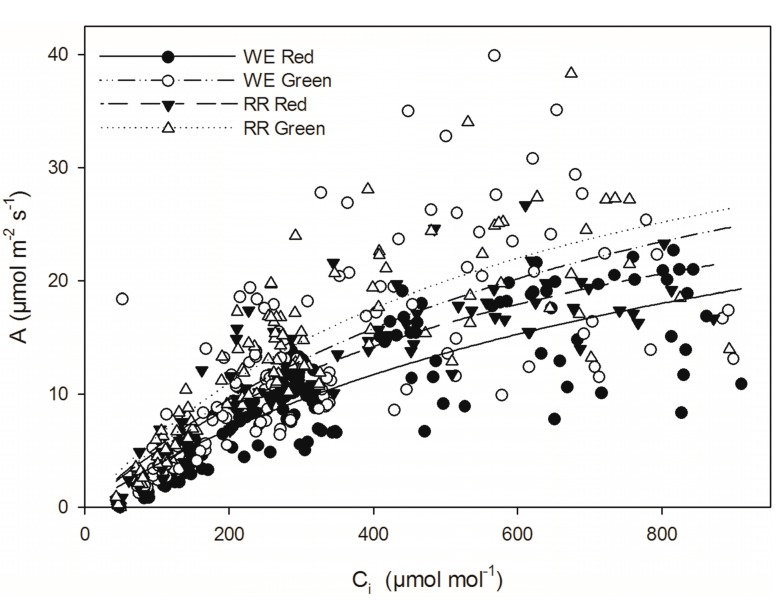
Photosynthesis (A) *versus* internal CO_2_ concentrations (C_i_) for red (solid symbols) *versus* green (open symbols) leaves at WE (circles) and RR (triangles). WE n_red_ = 16, n_green_ = 15; RR n_red_ = 12, n_green_ = 11.

A *versus* C_i_ curves measured during winter showed significantly greater carboxylation efficiency (CE) in green *versus* red leaves at both RR and WE sites (*p* < 0.05 for both sites individually, *p* < 0.01 when sites were combined; Figure 5 and Table 2). On average, CE in winter-green leaves was 30% higher than in winter-red leaves. Winter-green leaves also had higher maximum photosynthesis (A_max_) under saturating irradiance and CO_2_ than winter-red leaves at both study sites (Table 2); these differences were significant at WE (*p* < 0.05) and marginally significant at RR (*p* = 0.08). When data from both sites were combined, winter-green leaves had significantly (30%) higher A_max_ than winter-red leaves (*p* < 0.01). No differences in calculated stomatal limitation (I) or CO_2_ compensation point were observed between red and green leaves at either site (p_WE_ = 0.18, p_RR_ = 0.6, p_COMB_ = 0.36; p_WE_ = 0.71, p_RR_ = 0.72, p_COMB_ = 0.61 respectively).

**Table 2:** Mean values obtained from A/C_i_ curves at individual and combined sites. Data include: carboxylation efficiency (CE), relative stomatal limitation (I), carbon dioxide compensation point (CO_2_ CP), and A_max_ as maximum photosynthesis as measured at 1000 μmol·mol^−1^ [CO_2_] and saturating irradiance (μmol·m^−2^ s^−1^). Asterisks denote statistical significance between means (±SE) of red and green leaves (* *p* < 0.1, ** *p* < 0.05, *** *p* < 0.01).

	n	CE	I	CO_2_ CP	A_max_
Red WE	16	0.0475 ± 0.011 **	0.170 ± 0.071	49.0 ± 13	17.7 ± 4.4 **
Green WE	15	0.0649 ± 0.027	0.185 ± 0.15	47.4 ± 11	23.8 ± 8.3
Red RR	12	0.0613 ± 0.014 **	0.183 ± 0.079	41.1 ± 10	19.4 ± 3.0 *
Green RR	11	0.0757 ± 0.018	0.167 ± 0.059	39.8 ± 7.2	24.0 ± 6.6
Red AVG	28	0.0534 ± 0.014 ***	0.171 ± 0.076	45.6 ± 12	18.4 ± 3.9 ***
Green AVG	26	0.0694 ± 0.024	0.195 ± 0.11	44.2 ± 10	23.9 ± 7.5

### 2.4. Discussion

This study provides strong support for the photosynthetic-inferiority hypothesis for intraspecific leaf reddening during winter. According to this hypothesis, individuals with a reduced photosynthetic capacity (e.g., nitrogen-deficient individuals) synthesize anthocyanin pigments under high-light, cold-temperature (winter) conditions as a means of balancing energy capture with reduced demand. Specifically, we demonstrate that winter-red leaves exhibit significantly lower leaf N, reduced chlorophyll content, and lower photosynthetic capacity compared to co-occurring winter-green leaves, and also, that anthocyanin pigments attenuate a physiologically-significant portion of photosynthetically active radiation (PAR).

Red leaves of *L. japonica* contained significantly less (23% on average) leaf nitrogen than green leaves during winter at all three field sites (Figure 1A). These differences persisted during summer at the I40 site, which was the only field site where summer N measurements were made (Figure 1B). We suspect that reddening only manifested during winter due to the additional photoinhibitory stress imparted by cold temperatures [5,33]. These findings are consistent with previous studies reporting an inverse correlation between N content and anthocyanin synthesis in leaves within species [37,38,39,40,41,42]. Although determining the ultimate cause for the difference in nitrogen content between individuals examined was beyond the scope of this study, two anecdotal observations suggest that above-ground damage/defoliation may be responsible for reductions in leaf N, and consequent leaf reddening, in winter-red *L. japonica*. First, at RR, the portion of the slope featuring the highest density of winter-red individuals appeared to have been sprayed with an unknown, broad-spectrum herbicide during the summer following our measurements, resulting in complete necrosis of all above-ground plant matter; however, the area where winter-green individuals were present in higher frequency remained verdant (presumably not being sprayed). If this same spatial pattern of herbicide application also occurred at some point prior to our experiment, it could explain the reduction in N among the red-leafed individuals, as translocation of foliar nitrogen from leaves would not have been possible prior to their abrupt senescence. Similarly, a colleague anecdotally reported that pruning of above-ground *L. japonica* on one side of a walkway resulted in subsequent winter-reddening of new growth on the pruned side, but not the un-pruned side [48]. Regardless of the ultimate cause, significant reductions in foliar nitrogen were observed in winter-red individuals of *L. japonica* at all three field sites, and we believe that this is a proximate cause for the photosynthetically-inferior characteristics of winter-red individuals described below.

Ribulose-1,5-bisphosphate carboxylase/oxygenase (Rubisco) accounts for roughly 50% of photosynthetic N [43,49], resulting in a strong correlation between leaf N content and photosynthetic capacity [43,46,50]. Nitrogen deficiency is also known to correlate with a decrease in proteins involved in synthesis of chlorophyll, and chlorophyll *a/b* binding protein [44,51,52], which would limit the photon-capturing capacity of the photosystems and further reduce photosynthetic capacity. Consistent with these symptoms of N limitation, we demonstrate that winter-red *L. japonica* leaves exhibited significantly reduced photosynthetic capacity, carboxylation efficiency, quantum yield efficiency, and chlorophyll content relative to green-leafed individuals during winter. Specifically, A/C_i_ curves illustrate that red-leafed individuals exhibited significant (23% on average) reductions in maximum photosynthesis (A_max_) and carboxylation efficiency (CE) (23%) relative to green-leafed individuals (Figure 5, Table 2). Stomatal limitation did not significantly differ between red and green-leafed individuals in A/C_i_ curves, suggesting limitations to photosynthesis were biochemical. Similarly, diurnal measurements of photosynthetic gas exchange in the field during winter showed that red-leafed individuals generally exhibited significantly lower A_sat_ relative to green-leafed individuals, despite similar values of g (Figure 3C,D). Winter-red individuals also had lower total chlorophyll content per unit leaf area on average relative to green-leafed individuals both during summer (11% lower) and winter (40% lower) (Figure 2), as well as significantly reduced QYE and A_sat_ per unit leaf area (Table 1). These results corroborate previous reports demonstrating a photosynthetic inferiority (both in terms of reduced capacity for energy capture and processing) in winter-red individuals relative to green [40].

The significant reduction in photosynthetic capacity in N-deficient individuals provides a physiological basis for anthocyanin synthesis. As previously described, engagement of photoprotection has been shown to be inversely correlated with energy processing capacity [34,35]. Hence, an increase in anthocyanin content (which imparts photoprotective light-attenuating and antioxidant functions) would seem a suitable response for N-limited individuals [39]. Indeed, it has previously been demonstrated that N deficiency corresponds with up-regulation of expression of genes involved in synthesis of anthocyanin [44,53,54]. In another study, transcription levels of genes involved in the anthocyanin pathway were increased 7.6 to 49.2 fold under N-limiting conditions [55].

In order to assess whether anthocyanins attenuate a physiologically-significant amount of sunlight, light-response curves were derived using red/blue LED and compared to curves derived using ambient sunlight (Figure 4). This allowed for comparison of photosynthetic parameters with and without potential interference by the (green-light absorbing) anthocyanic layer. Consistent with our hypothesis, under red/blue LED, red-leaves exhibited a significant increase in quantum yield efficiency (QYE), and a significant reduced light compensation point (LCP) relative to measurements derived using ambient sunlight (Figure 4A and C). In green-leafed individuals, there was no significant difference in QYE or LCP under LED relative to ambient sunlight at WE, though significant (albeit substantially less dramatic) differences were observed at RR (Figure 4B and D respectively). When data from both field sites were combined, red leaves had a significant, 30% mean reduction in QYE under ambient sunlight compared to LED, while no significant difference was observed in green leaves. Similarly, red leaves exhibited a 180% higher LCP on average under sunlight compared to LED, while green leaves only presented a 68% increase, representing a three-fold difference between the two groups; both of these differences were statistically different.

## 3. Experimental Section

### 3.1. Plant Material and Field Sites

**Figure 6:**
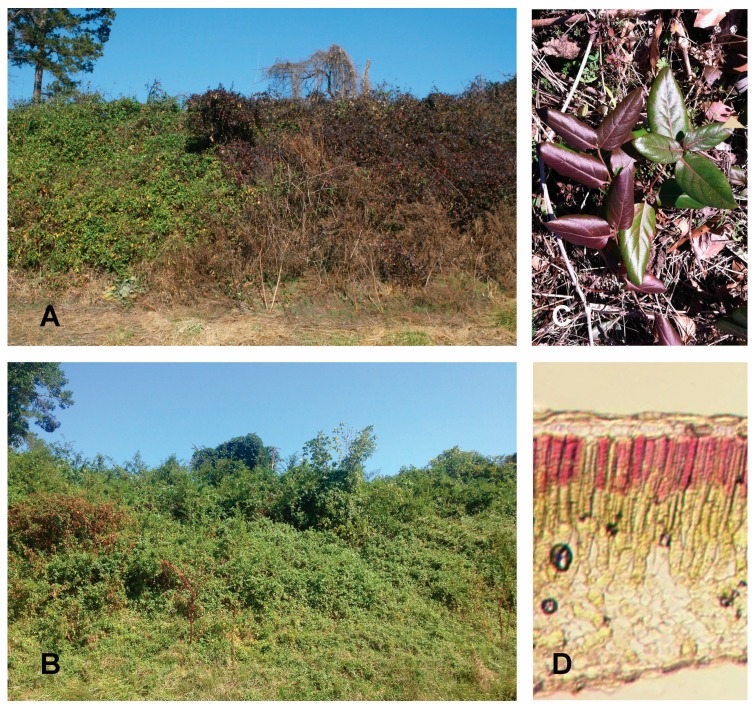
Photographs of *Lonicera japonica*. (**A**) I40 site during winter; green (**left**) and red (**right**) “color sides” are visibly distinct; (**B**) I40 site during summer; all *L. japonica* individuals presenting with green leaves; (**C**) Co-occurring red and green individuals of *L. japonica* as found *in situ* at RR; (**D**) Cross section of red *L. japonica* leaf, with anthocyanin pigments in the uppermost palisade layer.

*Lonicera japonica* Thunb. (Japanese honeysuckle) is an invasive vine found in the majority of the continental United States [56]. Three south-facing slopes in the Piedmont of North Carolina, USA featuring vines with both winter-red and winter-green leaves were utilized during this study. The Railroad site (RR) (36°10'21.24'' N, −80°26'31.83'' W) consisted of a fully-exposed, south-facing embankment located approximately 10 m from a roadside, situated along a railroad track. Red and green individuals co-occurred irregularly throughout the field site, although a distinct, uniformly green population occurred in one section of the embankment lying adjacent to a land-bridge overhanging a stream. All individuals were fully exposed to sun for >6 h per day during winter. The West End site (WE) (36°09'30.87'' N, −80°26'27.16'' W) consisted of two slopes, one southeast-facing, the other southwest-facing, in a residential area. The site was exposed to full sunlight during most of the day during winter, although presence of some evergreen trees resulted in brief, punctuated (1–2 h) shade intervals throughout the day. Red and green-leafed vines at this site were heavily intertwined, resulting in no clear definition between red and green populations. A third site along Interstate 40 Westbound (I40) (36°06'18.52'' N, −80°27'62.84'' W) was added in January 2013 for additional nitrogen and chlorophyll measurements (site pictured in Figure 6A,B). Field measurements at this site were limited due to the close (<5 m) proximity to high-speed vehicles, hence, no gas exchange measurements were made at this site. Red and green populations at I40 were distinctly separated, with green-leafed individuals being located primarily on the west side of the slope, and red-leafed individuals on the east side (Figure 6A).

In all sites, red and green leaves were similar in size, featured similar leaf orientations, and occurred in seemingly identical environments with respects to azimuth, sunlight exposure, and precipitation. Visible leaf reddening in *L. japonica* leaves began during mid-December, and remained until new leaves developed the following spring. All leaves used in this study were fully-developed, and were either distinctly red or green (e.g., Figure 6C); leaves with intermediate concentrations of anthocyanin were not used in this study.

To view anatomical distribution of anthocyanin pigments, sample red *Lonicera japonica* leaves were hand-sectioned and mounted on a Zeiss Axioplan upright microscope (Carl Zeiss Inc., Thornwood, NY, USA). Sections were viewed under bright-field microscopy, and images captured using a Hamamatsu C5810 three-chip cooled color CCD camera (Hamamatsu Photonics; Hamamatsu City, Japan).

### 3.2. Leaf Nitrogen

Red and green leaves were harvested from WE in February 2011, RR in January 2012, and I40 in January 2013. Since I40 showed dramatic spatial separation of red and green populations, measurements could be made on winter-red *versus* winter-green sides of the slope during the summer as well (August 2013) when both “color sides” were green. Five to seven shoots (4–6 leaves per shoot) of each phenotype were randomly sampled from all sites on each measurement date. Leaf tissues were stored briefly in wet paper towels, then (within 2–3 h) homogenized in liquid nitrogen using a mortar and pestle, and oven-dried at 60 °C. Percent leaf nitrogen was quantified using a CHN 2400 Elemental Analyzer (Perkin Elmer Corporation, Norwalk, CT, USA). A NIST (National Institute of Standards and Technology) standard was also run every 22 samples to ensure accuracy of measurements. Normality was assessed using the Shapiro-Wilk test using JMP (3.2.2) statistical package (SAS Institute Inc., Cary, NC, USA) with normality defined as *p* > 0.05. Means were compared within individual sites using a one-tailed Student’s *t* test in Microsoft Excel (14.3.5) (Microsoft Corporation, Redmond, WA, USA). Data sets from the three sites were also combined and analyzed using a randomized complete block design ANOVA using Statistix (9.0) (Analytical Software, Tallahassee, FL, USA).

### 3.3. Chlorophyll Content

Fresh leaf tissues were collected from RR, WE, and I40 sites on the morning of 10 January 2013 and immediately transported to the laboratory in a wet paper towel within a plastic bag. Five replicates of each colored leaf from each site were obtained (30 samples total). Additionally, on 27 August 2013 (when all plants were green), six leaves from each “color side” of I40 were collected and analyzed. For all assays, three 0.317 cm^2^ hole-punched tissue sections were excised from each leaf, and immediately extracted in 3 mL *N,N* dimethylformamide in the dark at room temperature for 24 h. The absorbance of the supernatant was then determined spectrophotometrically (Ocean Optics, USB4000-UV-VIS with USB-ISS-UV/VIS attachment, Dunedin, FL, USA). Chlorophyll *a* and *b* were estimated on a per unit leaf area basis using equations from Porra [57]. Data were tested for normality via Shapiro-Wilk test using JMP (3.2.2). Means within individual sites were compared using a two-tailed Student’s *t* test in Microsoft Excel (14.3.5), and randomized complete block design ANOVAs were run using Statistix (9.0) for combined data sets.

### 3.4. Photosynthetic Gas Exchange

Diurnal photosynthetic gas exchange measurements for red and green-leafed individuals were made using a Li-Cor 6400XT (Li-Cor, Lincoln, NE, USA) with Li-6400-02B red/blue LED chamber with PAR (photosynthetically active radiation) set to 1500 μmol·m^−2^·s^−1^ during one warm (low temp > 0 °C), mostly sunny winter day at RR (13 January 2012) and WE (6 February 2011). Measurements were collected at WE at 1000, 1200, 1500, and at RR at 0900, 1200, and 1600. During all measurement intervals, 3–15 individuals of each leaf color were sampled, with measurements alternating randomly between leaf color.

Photosynthetic light-response curves (LRC) were derived on warm (low temp > 0 °C), sunny days during the winters of 2011–2013 using a Li-Cor 6400XT. Curves were derived separately using either ambient sunlight (Li-6400 standard clear-top chamber plus neutral-density shade films) or the red/blue LED light source. Curves were derived both with sunlight and red/blue LED in order to compare the light response of photosynthesis with and without the putative light-attenuating effects of anthocyanins, which absorb strongly in the green wavelengths. Separate leaves were chosen at random for each light-response curve. Within individual days, measurements alternated between red and green leaves. The light-source used in light-response measurements was randomized between days. Measurements were taken at ambient temperature and humidity, between 0700 and 1330. For LRC utilizing the LED light source, each curve was initiated at 2000 μmol·m^−2^·s^−1^ and was subsequently decreased incrementally in a total of 12 stepwise reductions until 0 μmol·m^−2^·s^−1^ was reached. The same protocol was used for LRC utilizing ambient light and neutral density shade screens, though maximum PAR values ranged from 1031 to 1799 μmol·m^−2^·s^−1^. For each curve, the following parameters were determined: dark respiration rate (DR), determined as CO_2_ flux at 0 μmol·m^−2^·s^−1^, light compensation point (LCP), the level of PAR corresponding with 0 μmol·m^−2^·s^−1^ net CO_2_ uptake, quantum yield efficiency (QYE), estimated as the slope of the linear, light-limited portion of the curve between 0 and 200 μmol·m^−2^·s^−1^ PAR, and photosynthesis at saturating irradiance (A_sat_).

Photosynthetic CO_2_ response (A/C_i_) curves were derived using Li-Cor 6400XT, equipped with Li-6400-02B red/blue LED light source set at saturating irradiance (1500 μmol·m^−2^·s^−1^). Measurement protocol (with regards to randomization and replication) was similar to that described above for LRC. Measurements were made between 0700 and 1330 under ambient temperature and humidity conditions. Measurements began near ambient [CO_2_] levels (400 μmol·mol^−1^), then decreased incrementally in five stages to 50 μmol·mol^−1^, returned to 400 μmol·mol^−1^, and then increased in three increments until 1000 μmol·mol^−1^ was reached. Measurements were taken after allowing approximately 60–90 s following each CO_2_ adjustment to allow leaves to acclimate. For each curve, carboxylation efficiency (CE), relative stomatal limitation (I), and CO_2_ compensation point (CO_2_ CP) were determined as described by Ku and Edwards [58], Farquhar *et al.* [59], and von Caemmerer and Farquhar [60].

All photosynthetic gas exchange data were tested for normality as previously described. Red *versus* green diurnal measurements were compared at each time point within each site individually using a two-tailed Student’s *t* test, using Microsoft Excel (14.3.5). Parameters derived from light response curves were analyzed by comparing red *versus* green-leaf values under each light source, and LED *versus* sunlight values according to leaf color. Within sites, mean DR, LCP, QYE, and A_sat_ for were compared using a two-tailed Student’s *t* test, and a randomized complete block design ANOVA via Statistix (9.0) was used to analyze combined-site data. For A/C_i_ curves, mean CE, I, CO_2_ CP and A_max_ values for red *versus* green leaves were compared within each site individually using a two-tailed Student’s *t* test, and combined using a randomized complete block design ANOVA via Statistix (9.0).

## 4. Conclusions

Results presented here support the photosynthetic-inferiority hypotheses for intraspecific winter-leaf reddening, which posits that winter-red leaves suffer from reduced nitrogen, photosynthetic capacity, and/or chlorophyll content. Furthermore, we demonstrate that anthocyanin pigments attenuate a physiologically-significant amount of green-light.

The association between winter-reddening and photosynthetic deficiency could potentially serve as a useful diagnostic for identifying photosynthetically-inferior individuals within a population, which could have valuable applications to crop management (e.g., see [61,62]). We are hesitant, however, to extend these results to explain interspecific differences in leaf reddening at the community level. Previous studies comparing co-occurring winter-red and winter-green species have yet to demonstrate significant differences in photosynthetic capacity or leaf N [16,18,63]. It is likely that confounding differences in anatomy and/or physiology between different species make such comparisons difficult, though our results combined with those of previous studies certainly encourage further investigation of this hypothesis at the community level.
